# Parental reports on language development in toddlers and preschoolers based on the Croatian version of Communicative Development Inventories III

**DOI:** 10.3389/fpsyg.2023.1188550

**Published:** 2023-07-20

**Authors:** Lidija Šmit Brleković, Jelena Kuvač Kraljević

**Affiliations:** ^1^Postgraduate Doctoral Programme Speech, Language and Hearing Disorders, Faculty of Education and Rehabilitation Sciences University of Zagreb, Zagreb, Croatia; ^2^Department of Speech and Language Pathology, Faculty of Education and Rehabilitation Sciences University of Zagreb, Zagreb, Croatia

**Keywords:** MacArthur-Bates Communicative Development Inventories, parental reports, vocabulary, grammar, metalinguistic awareness, language development, Croatian

## Abstract

**Introduction:**

Previous studies have focused on understanding parental attempts to record language development in children, across many typologically different languages. However, many of these studies restricted their assessment to children up to the age of 3 years. The aim of this paper was to move this boundary by examining language development in typically language developed children older than 3 years.

**Methods:**

Using the Croatian version of the Communicative Development Inventories III (CDI-III-HR), we investigated the contribution of parental reports of a child’s lexical, grammatical, and metalinguistic awareness abilities to general language abilities assessed by clinicians. Participants included the parents of 151 children between the ages of 30 to 48 months, who completed the CDI-III-HR and reported on their child’s language abilities.

**Results:**

Our results show that age is significantly associated with the lexical, grammatical, and metalinguistic awareness abilities of a child’s language development. These findings confirm that all three abilities increase with age and that parents can perceive changes in a child’s language development. The subsections of CDI-III-HR were moderately to strongly associated with each other, with the strongest association being between lexicon and grammar, suggesting that they remain closely related after the age of 30 months. Parental assessments of a child’s language development are a better predictor of language production than language comprehension, with grammar making the most consistent and significant contribution.

**Discussion:**

This study confirms that the development of grammatical abilities is the most prominent skill between the ages of 30 to 48 months and that parents can observe the transition in the child’s language development through their usage of grammar in words to grammar in sentences. Based on the selected sample of children, we discovered different patterns of parental success in assessing the child’s language ability. These findings indicate that parents can act as valuable sources of information regarding the child’s language abilities, but clinical assessments of early language development should consider many other formal sources of information in addition to parental reports.

## Introduction

The discussion regarding the optimal method for effectively assessing infant and toddler language development continues to be the subject of intense debate in the areas of developmental psychology and speech-language pathology for two main reasons. First, none of the methods developed so far have been able to capture the multidimensional nature of language. Second, each method has its own shortcomings (Dockrell, 2001): for example, standardised measurement instruments are very often not an appropriate method for assessing children’s language in the early years of life, primarily because it is difficult to ensure the child’s cooperation in a new environment and with a new person (Law and Roy, 2008).

These shortcomings can be compensated to some extent by parental reports. They have been proved to be increasingly useful as a good initial method of describing a child’s language and communication abilities. Additionally, parents spend a considerable amount of time with the child and they are the most frequent interlocutors during the early years of the child’s life. This allows them to observe the child’s language abilities under natural conditions, as well as in a wide range of situations (Guiberson et al., 2011). Parental reports can be a valuable source of initial information, especially in cases where observational data indicate concerns about language and communication development.

Likewise, parental reports also have certain limitations. Parents may be biased and may overestimate or underestimate the child’s abilities due to various clinical, educational, and social circumstances (Feldman et al., 2000; Law and Roy, 2008). For example, Jackson-Maldonado et al. (2013) showed that mothers with lower levels of education were more likely to overestimate their child’s level of word understanding, especially during the early phase of development, while mothers with higher levels of education were more likely to apply a higher standard when interpreting the ability of early understanding, resulting in lower average scores in this sample of children. These limitations raise the question of the reliability of parental assessment of the child’s language in the late toddler and preschool periods.

### Parental assessment of language development of children older than 30 months using MB-CDI-III

Over the past 50 years, researchers and clinicians have widely recognised and confirmed the ecological validity of standardised measures of parental reports. Parental reports of a child’s language and communication abilities have become an integral part of screening and diagnostic procedures. Therefore, many standardised measurement instruments are available in order to collect specific data from parents, such as the Age and Stages Questionnaire (Bricker and Squires, 1999) or the Language Development Survey (LDS; Rescorla, 1989). However, only a few of these are designed to assess the language development of children who are more than 3 years old. For example, the Language Use Inventory (LUI; O'Neill, 2007) can be used to assess language abilities, but it is focused exclusively on specific language aspects, in this case, pragmatics.

Fenson et al. (2007) developed the MacArthur-Bates Communicative Development Inventories III (MB-CDI-III) to assess lexical and syntactic development in children aged 30 to 37 months. This version is an extension of the MacArthur-Bates Communicative Development Inventories (MB-CDI, Fenson et al., 1990, 2007). MB-CDI was originally developed in order to conduct parental assessments of broader aspects of language and communication abilities in children up to 30 months of age. This is a period during which language development is slow, thus ensuring that parents perceive the qualitative changes in the child’s language. Using the MB-CDI, Dale et al. (1989) demonstrated that parental reports were accurate, valid, and reliable when parents were limited to reporting current and novel behaviors, as well as when reporting was based on a recognition format.

The MB-CDI-III consists of three subsections: vocabulary checklist (100 items), syntactic complexity (12 items), and language use (12 items). It was validated based on a sample of 356 children aged 30 to 37 months. However, ceiling effects occurred in later months covered by the instrument (34–37 months), particularly in the syntactic complexity and language use subsections (Fenson et al., 2007). Instrument showed good concurrent validity. In a study of the validity of parental reports of the language abilities of children aged 2 and 3 years, Feldman et al. (2005) found a statistically significant, moderate correlation between the scores on all three subsections of the MB-CDI-III for children aged 3 years and the scores obtained using two standardised tests: The McCarthy Scales of Children’s Abilities (*r* = 0.47–0.56, *p* < 0.001) and Peabody Picture Vocabulary Test – Revised (*r* = 0.41–0.49, *p* < 0.001). In addition, the authors reported statistically significant but low correlations between the three subsections and conversational language measures in children at age three (*r* = 0.26–0.42, *p* < 0.001).

Given the many ceiling effects that the MB-CDI-III (Fenson et al., 2007) showed, especially in the upper half of the age range studied, Eriksson (2017) revised this assessment by introducing four semantic categories (food words, body words, mental words, and emotion words) into the vocabulary subsection, adding a new subsection on metalinguistic awareness, and extending the use of the instrument to 48 months of age. To our knowledge, the MB-CDI-III has been developed in nine languages (Table 1). Based on the details listed in Table 1, it can be seen that the adaptation of this instrument has been carried out in two directions – first, according to the original American inventory (as in Basque or Hungarian), and second, according to the revised Swedish inventory (as in Croatian and Estonian). Regardless of whether the languages follow the American or Swedish version, there are differences in the structure or the number of items in the newly developed versions of the CDI-III. For example, in the Portuguese inventory, there is no metalinguistic awareness subsection, or in the Estonian inventory, there are six items in pronunciation, while the Swedish inventory has only one. Third, although the instrument has broad applicability in language assessment of different populations (clinical groups, bilingual speakers, children from different social backgrounds), the authors’ different original motivations for developing the instrument in one language are also evident. The Hungarian inventory, for example, was developed for screening children experiencing language delays. On the other hand, the Norwegian authors strongly emphasise the importance of the Norwegian CDI-III for multilingual speakers.

**Table 1:** Overview of the CDI-III (data taken from Cadime et al., 2021 and supplemented with recent data).

Reference	Language	Age range of target population (years)	Subsections and items				
			Vocabulary	Syntax and morphology	Uses of language	Metalinguistic awareness	Additional items
Fenson et al. (2007)	English (American) Original version	2.6–3.0	Word checklist (100)	(a) Syntactic complexity (12) – Pairs of sentences with varying complexity (parents must flag one in each pair that most resembles what the child says)	Using language (12) – Questions on different language uses with a yes/no response	–	(a) One question on whether the child already combines words (not yet/sometimes/often) (b) Mean length of utterances –Parents must list the three longest sentences that they heard from their child recently
Guiberson and Rodríguez (2010) ^*^	Spanish (Pilot INV–III)	3.0–5.2	Word checklist (100)	(a) Syntactic complexity (12) - Pairs of sentences with varying complexity (parents must flag one in each pair that most resembles what the child says)	Using language (12) – Questions on different language uses with a yes/no response	–	(a) One question on whether the child already combines words (not yet/sometimes/often) (b) Mean length of utterances –Parents must list the three longest sentences that they heard from their child recently
Garcia et al. (2014)	Basque	2.6–4.2	Word checklist (120)	(a) Syntactic complexity (29) – Pairs of sentences with varying complexity (parents must flag one in each pair that most resembles what the child says) (b) Morphology – one list of suffixes (16) and one list of verbs (20) (parents should indicate the ones produced by their child)	Using language (10) – Questions on different language uses with a yes/no response	–	(a) One question on whether the child already combines words (not yet/sometimes/often) (b) Mean length of utterances –Parents must list the three longest sentences that they heard from their child recently
Eriksson (2017)	Swedish (SCDI-III)	2.6–4.0	Word checklist (100) divided into four semantic categories: food words (16), body words (26), mental words (30), emotion words (28)	(a) Language complexity (10) –Pairs of sentences (parents must indicate if the child uses one of them or if both are used equally) (b) Grammar (8) – questions on the use of grammar markers with a three-category frequency response scale: never/sometimes/everyday	–	Metalinguistic Awareness (7) – Questions with a yes/no response to assess phonological and orthographic awareness, as well as the awareness of the existence of other languages	(a) One question on children’s general level of communication with six alternatives (e.g., not yet talking) (b) Pronunciation – one question on how the child sounds compared to other children of the same age, with three response alternatives: like an age-mate, a younger, or an older child.
Garmann et al. (2019)	Norwegian Pilot	2.6–4.0	Word checklist (100) divided into four semantic categories: food words (16), body words (26), mental words (30), emotion words (28)	(a) Language complexity (10) –Pairs of sentences (parents must indicate if the child uses one of them or if both are used equally) (b) Grammar (8) – questions on the use of grammar markers with a three-category frequency response scale: never/sometimes/everyday	–	Metalinguistic Awareness (7) – Questions with a yes/no response to assess phonological and orthographic awareness, as well as the awareness of the existence of other languages	(a) One question on children’s general level of communication with six alternatives (e.g., not yet talking) (b) Pronunciation – one question on how the child sounds compared to other children of the same age, with three response alternatives: like an age-mate, a younger, or an older child.
Tulviste and Schults (2020)	Estonian (ECDI-III)	2.6–4.0^**^	Word checklist (101) divided into four semantic categories: food words (17), body words (26), mental words (30), emotion words (28)	(a) Language complexity (10) –Pairs of sentences (parents must indicate if the child uses one of them or if both are used alternately) (b) Grammar (7) – questions on the use of grammar markers with a three-category frequency response scale: never/sometimes/everyday	–	Metalinguistic Awareness (7) – Questions with a yes/no response to assess phonological and orthographic awareness	(a) One question on children’s general level of communication with six alternatives (e.g., not yet talking) (b) Pronunciation – one question on how the child sounds compared to other children of the same age with three response alternatives: like an age-mate, a younger, or an older child. Five items with a yes/no response to assess specific pronunciation difficulties
Cadime et al. (2021)	European Portuguese (CDI-III-PT) Pilot	2.6–4.0	Word checklist (166) divided into four semantic categories: food words (37), body words (34), mental words (45), emotion words (50)	(a) Syntactic complexity (26) – checklist presenting examples of syntactic structures (parents must indicate yes/no if the child produces the target structure)	–	–	–
Kas et al. (2022)	Hungarian (HCDI-III)	2.0–4.2	Word checklist (124)	(a) Syntactic complexity (12) – Pairs of sentences with varying complexity (parents must flag one in each pair that most resembles what the child says) (b) Morphology – one list of productive errors (12) (parents should indicate the ones produced by their child and have the possibility to add their own examples)	Using language (14) – Questions on different language uses with a yes/no response. Also completed by adding two questions related to children’s use of specific morphologically complex forms for asking for permission and for expressing conditional intentions.	–	(a) One question on whether the child already combines words (not yet/sometimes/often) (b) Example Sentences section (Mean length of utterances) –Parents must list the three longest sentences that they heard from their child recently
Kuvač Kraljević et al. (n.d.)	Croatian (CDI-III-HR)	2.6–4.0	Word checklist (100) divided into four semantic categories: food words (16), body words (26), mental words (30), emotion words (28)	(a) Language complexity (10) –Pairs of sentences (parents must indicate if the child uses one of them or if both are used equally) (b) Grammar (8) – questions on the use of grammar markers with a three-category frequency response scale: never/sometimes/everyday	–	Metalinguistic Awareness (9) – Questions with a yes/no response to assess phonological and orthographic awareness, as well as the awareness of the existence of other languages	(a) One question on children’s general level of communication with six alternatives (e.g., not yet talking) (b) Pronunciation – one question on how the child sounds compared to other children of the same age, with three response alternatives: like an age-mate, a younger, or an older child. Five items with a yes/no response to assess specific pronunciation difficulties

^*^Pilot INV–III is not an endorsed adaptation of the CDI–III, it is a translated version of the CDI–III. ^**^So far, the study has collected data for children aged 2.10to 3.3 years (*N* = 100), but the authors aim to collect data for children in a broader age range.

All previously published CDI-III, developed according to the American or Swedish versions, reported relatively good intercorrelation between their subsections – vocabulary, grammar, and metalinguistic awareness. For example, the Hungarian CDI-III (HCD-III; Kas et al., 2022) was assessed based on a sample of 1,424 children between the ages of 24 to 50 months and showed that all variables except one – production error – were highly correlated with each other (for example, correlation between vocabulary and sentence was *r* = 0.956, *p* < 0.01). In the Swedish CDI-III (SCDI-III; Eriksson, 2017), all three subsections were significantly related to each other (*r* = 0.544–0.780, *p* < 0.01). The Estonian CDI-III (ECDI-III; Tulviste and Schults, 2020) was validated based on a sample of 100 Estonian-speaking children between the ages of 34 to 39 months. In this case, strong correlations were observed between vocabulary, grammar usage, and sentence complexity (*r* = 0.71–0.88, *p* < 0.001), but the correlation between phonological and orthographic awareness and other components was weak (*r* = 0.21–0.42, *p* < 0.05). In the European Portuguese adaptation of CDI-III (CDI-III-PT; Cadime et al., 2021), there were positive strong correlations between vocabulary and grammar (*r* = 0.659, *p* < 0.001). In Basque CDI-III (Garcia et al., 2014), assessed on a sample of 1,024 children between the ages of 30 to 50 months, all subsections were highly correlated with each other (*r* = 0.59–0.81, *p* < 0.001). In the Norwegian pilot study, a moderate correlation was found between level of communication on the one hand and vocabulary (*r* = 0.483, *p* < 0.01), grammar (*r* = 0.496, *p* < 0.01), and pronunciation (*r* = 0.449, *p* < 0.01) on the other hand. Considering these correlations with level of communication, the authors believed that it is sufficient to ask parents how their child speaks, without delving deeply into lexical and grammatical development (Garmann et al., 2019).

Among all new developed CDI-III, only four reported concurrent validity, i.e., validity that shows the extent of agreement between assessments conducted at the same time. In ECDI-III (Tulviste and Schults, 2020), concurrent validity was established with positive medium correlations between the ECDI-III components and the Estonian version of the New Reynell Developmental Language Scales subsections (*r* = 0.43–0.65, *p* < 0.001) and ECDI-II components (*r* = 0.52–0.87, *p* < 0.001). In CDI-III-PT (Cadime et al., 2021), medium to strong correlations were observed between CDI-III-PT scores and the language subsection of The Griffiths Mental Development Scales (Luiz et al., 2006). In the Basque CDI-III (Garcia et al., 2014), a medium partial correlation was found controlling for age between the components of a Basque CDI-III and the Peabody test (Dunn et al., 2006) (*r* = 0.60–0.72, *p* < 0.001). In a Norwegian pilot study (Garmann et al., 2019), 28 children were recorded participating in a 30-min spontaneous conversation and significant moderate to strong correlations were observed between the words from CDI-III and two measures related to a child’s spontaneous conversation abilities: the number of different words and mean length of utterances (MLU).

### Language development in children between 2 and 4 years of age

After the age of 2 years, language abilities in children increase exponentially at all levels: not only quantitatively, but also qualitatively, in terms of complexity and the depth in the conceptual level of lexical units. For example, at 2 years, a child’s lexicon contains about 250 words, while at 3 years, the lexicon contains about 1,000 words, and at 4 years about 1,600 words (Owens, 2020). Besides open-class words – nouns, verbs, and adjectives – two-year-olds, especially those with larger lexicons, have usually begun to acquire closed-class words such as articles, pronouns, prepositions, question words, and quantifiers, which are used to express grammatical meaning in sentences (Stolt, 2018). However, three-year-olds possess lexicon with more abstract words that represent different mental states. These words differ in their semantic-conceptual properties and are therefore more demanding to acquire for several reasons: their perceptual properties in relation to the referent in the child’s environment are not so transparent and direct, which means that the child cannot rely on context to interpret the meaning of such words, but they have to extract it from an abstract concept. All this affects the later appearance of these words in the child’s lexicon. While most concrete words such as action verbs like *walk*, *cook*, or *drink*, appear before a child’s second year of life, abstract mental verbs do not appear until the third year. Some of them, especially those that are very similar in meaning (e.g., *think* and *know*), remain obscure to children until the fourth year of life (Papafragou et al., 2007).

Greater lexical knowledge is the trigger for the master of a greater number of morphological rules, and consequently, a bigger lexicon and more advanced morphology are the trigger for the production of more complex and longer syntactic structures. This relationship between expressive lexical ability and grammatical development has been reported in many monolinguistic studies in different languages (Maital et al., 2000; Stolt et al., 2009), as well as in cross-linguistic studies (Thordardottir et al., 2002; Devescovi et al., 2005; Kuvač Kraljević et al., 2021). Moreover, a cross-linguistic study involving Croatian, Estonian, and Finnish (Kuvač Kraljević et al., 2021) confirmed that two-year-old children who combined words had lexicon approximately four times larger than those who still had not yet started to combine at the same age, suggesting that lexical development can predict syntactic development. However, the trajectory of lexical and grammatical development is not always monodirectional in a way that only lexical abilities support and predict grammatical development. It is more correct to say that the relationship between grammatical and lexical development is bidirectional. As the child begins to use the grammatical system productively, it facilitates lexical growth, i.e., as the lexicon grows, it increasingly supports grammatical development (Moyle et al., 2007). The interplay between these two skills varies across the different years. Progress in the development of both abilities can sometimes occur simultaneously, but sometimes, especially in the first years of life, it can occur sequentially, which means that the development of one of these abilities is not linear (Bates and Goodman, 2001).

By age three, children begin to master many grammatical forms, but they continue to build on some that they acquired in their second year of life (for example, they continue to use negation (Tager-Flusberg, 2001), but also learn to incorporate negation into more complex forms (Reed, 2017)). Generally, after age three, children ask more and more questions and have better control on sentence-internal features such as predicate-subject-object agreement or independent and dependent relations. However, they also increase production on the discourse level through better mastering of across-sentence features. For example, although three-year-old children exhibit a lack of contextual information about the time, place, and chronological order of events when recounting a personal story (Reese et al., 2011), they can produce and tell a short story with two events (Peterson and McCabe, 1983). In short, although the period from 2 to 4 years is very variable in terms of pace and style, this period represents the stage of language development when children extend the grammar of words to sentences.

It is certainly important to observe language development from the perspective of receptive and expressive language, that is, from the connection between comprehension and production. Despite the widespread assumption that comprehension is always more advanced than production, differences in the relationship between comprehension and production in early language development are much more common than assumed. Many studies report a positive, significant, small to moderate correlation between comprehension and production, as seen in Bornstein and Hendricks’s (2012) study. However, this correlation is not as simple as it first appears. For example, in a large sample of 101,250 children, ages 2;00 to 9;11, from sixteen under-researched languages, the authors found that the mean of comprehension and production varied with the child’s age, reaching an asymptote at age 5;00. Thus, comprehension does not always predict production (Bauer et al., 2002). In addition, production has been found to precede comprehension for certain language phenomena, such as word order, verbal inflection or object pronouns (Hendriks, 2014). Bates (1993) also describes a dissociation between comprehension and production for typically language developing children but also in some clinical groups such as late talkers, who show enormous variability in receptive language abilities, from typical to impaired. Thus, comprehension and production can also be sometimes defined as ‘dissociated’ processes (Bates, 1993; Bauer et al., 2002) during language development.

After the age of three, children slowly begin to think about language not only as an object of knowledge, but also as a means of communication (Sinclair, 1986). This metalinguistic ability develops gradually and includes the knowledge that language consists of more discrete elements (e.g., phonemes, syllables, words) and can also be represented in written form. Aside from a better understanding of the inherent nature of language, metalinguistic awareness is mandatory for reading and writing at all stages of its development – from emergent and beginning to conventional literacy. Emergent literacy refers to literacy development that originates in early childhood, i.e., before the onset of formal conventional literacy instruction. Whitehurst and Lonigan (2001) emphasised that, in addition to oral language, phonological and orthographic awareness act as precursors to early literacy that correlates most strongly with later conventional reading. Phonological awareness refers to a child’s ability to identify smaller language units of words such as phonemes and syllables (Anthony and Francis, 2012). Knowledge of the alphabet is one of the best predictors of reading achievement in school (Whitehurst and Lonigan, 2001). In addition to being directly related to decoding written language, letter knowledge also has an impact on phonological awareness. Another pathway of print awareness and letter knowledge is through writing, and this is called orthographic awareness. Behaviors such as pretend writing or learning to write one’s own name are examples of emergent writing. Since parental linguistic input plays an important role in the development of the child’s spoken language abilities, it can be assumed that it is also important in promoting metalinguistic awareness. For example, shared book reading, an activity that is part of the family literacy construct, plays a special role in the development of spoken language, especially in lexical development (Blewitt and Langan, 2016), but also in the development of emergent literacy (Justice and Kaderavek, 2002). Finally, family literacy, defined by the frequency and quality of shared reading, which can be summarised as a cultural practice and the number of books at home as an indicator of cultural capital, is known to be a good predictor of children’s early and later literacy (Niklas et al., 2020).

All these determinants of language between 2 and 4 years of age – the more advanced vocabulary, complex syntax, and beginning to think about language on a metalinguistic level – raises a logical question: how do parents observe and perceive such language development, given its quantity and content? With this framework in mind, the aim of this study is to investigate the concurrent validity of CDI-HR-III by analysing and comparing parental reports on the lexical, grammatical, and metalinguistic awareness abilities of typical developing children aged 30 to 48 months with data of general language abilities of the same children based on assessments made by clinicians.

The specific objectives of the study are:

1. to examine the influence of age on lexical, grammatical, and metalinguistic abilities of children,
2. to examine the interrelationships between the different subsections of the parental report,
3. to investigate the individual contributions of lexical, grammatical, and metalinguistic awareness variables based on parental reports of the child’s general language abilities, and
4. with regard to the two assessment methods – parental report and formal assessment – to examine the agreement of the language performances of those children whose language achievement were in the lower range of average performance.

## Materials and methods

### Participants and procedure

Study participants included parents of a total of 173 children, who completed the CDI-III-HR and reported on their child’s lexical, grammatical, and metalinguistic abilities. Participants were recruited by speech and language pathologists (SLP) who work in the kindergartens attended by their children. After receiving consent from the parents who were willing to participate in the study, the SLPs explained how to fill out the CDI-III-HR. The participants then filled out the inventory themselves and returned the reports to the SLPs after a few days.

Once the SLPs received the completed inventories from the parents, they tested each child on the Comprehension and Production Scales of the New Reynell Developmental Language Scales (NRDLS-HR; Edwards et al., 2019) in their offices in the respective kindergartens. The analysis of the obtained data on the NRDLS-HR revealed that 22 children had below average performance on both scales – Comprehension and Production (standard scores ≤80 correspond to the 10% population of children with lowest achievement in language; see Norbury et al., 2016; Wu et al., 2023) – and were therefore excluded from further analysis. Therefore, in this study we included only those children whose NRDLS-HR scores exceeded the established threshold (standard scores ≥81) i.e., those whose language performance falls within the range of typical language abilities. The distribution of the scores obtained by the children included in the study (*n* = 151) on both NRDLS-HR scales are listed in Table 2.^1^

**Table 2:** Distribution of NRDLS-HR scores obtained by the children included in the study.

Scale	Standard score		
	81–90	91–100	> 100
NRDLS-HR Comprehension scale	9	38	104
NRDLS-HR Production scale	22	20	109

For the final analysis, 151 children included in the study were stratified into three age groups: youngest – from 30 to 35 months (*n* = 51; 30 girls and 21 boys), middle – from 36 to 41 months (*n* = 42; 21 girls and 21 boys), and oldest – from 42 to 48 months (*n* = 58; 29 girls and 29 boys). Of the total number of children, 31% lived in the eastern part of Croatia, 29% from the Adriatic part, and 40% from the central and northern part where one/third of the entire Croatian population lives (according to the last census, Croatian Bureau of Statistics, 2021). For 96% of the children, the CDI-III-HR was completed by their mother, while for 3% of the children, it was completed by their father and for 1% of the children, by both parents together.

The study and its protocol were approved by the Ethics Committee of the Faculty of Education and Rehabilitation Sciences, University of Zagreb (approval number: 251–74/22–01/2). After guaranteeing their anonymity and dignity, informed consent was obtained from all participants. In order to collect data on language development of children from different parts of Croatia, a total of 24 SLPs participated in this study. The requirement for SLP involvement was that he or she had to have a license to use NRDLS-HR. The inclusion of SLPs in the study, the recruitment of participants at daycare centers, and the entire testing procedure were approved by the Ministry of Science and Education (MSE 533–06-21-0002).

### Adaptation of the CDI-III in Croatian

The adaptation of CDI-III-HR (Kuvač Kraljević et al., n.d.) is a continuation of the adaptation of the MB-CDI I and MB-CDI II (Kovačević et al., 2007) and the short versions of the same inventories (Kuvač Kraljević et al., 2023). The adaptation of CDI-III in Croatian began in September 2018 after receiving approval from the CDI Advisory Board and Mårten Eriksson (University of Gåvle). The Croatian version of CDI-III follows the Swedish version (Eriksson, 2017), after taking into account the peculiarities of the Croatian language, especially in the grammar part. Croatian belongs to the group of South Slavic languages, and it is highly morphologically developed with seven cases, three genders, and two numbers in the noun system, as well as seven verbal classes based on infinitive and present tense forms (Barić et al., 1997).

The CDI-III-ḪR is an instrument for parents in which they are asked to mark the words they recognise in their children’s current spoken language. In addition, questions about the use of grammatical markers and the presence of metalinguistic awareness only had to be answered with *yes* or *no*. The first version of CDI-III-HR was developed in 2019 and consisted of 100 words taken from the Swedish version, with the same number of grammatical items. However, sometimes it was necessary to find suitable and equivalent substitutes for Swedish, and so completely new items were developed, reflecting the peculiarities of the Croatian language (such as verbal aspect or noun inflection). For the development of the items in the grammar and sentence complexity subsections, we performed a comprehensive review of available Croatian literature and developed the items based on empirical evidence (for example, Kovačević et al., 2009; Radić Tatar, 2013). These studies have shown that children at age of three and four mark all tenses in Croatian, include verb aspects, use more complex prepositional markers, compare adjectives, form interrogative clauses, use negation and conjunctions, as well as overgeneralise morphological rules. CDI-III-HR consisted of a list of words and pairs of sentences of different complexity.

In May 2020, we asked 45 parents of children between 30 and 48 months of age, who were also trained SLPs, to complete the CDI-III-HR and evaluate its efficacy using both parental and professional knowledge. These parents provided two types of feedback: (1) linguistic – for example, that it is necessary to give more examples for several items in the grammar subsection and (2) technical – refers to graphical solutions of subsections. For example, the structure of the sentence complexity subsection was confusing for many parents. That is why it was restructured in a way that simple and complex sentences that form a pair are listed one below the other on the left side and the scoring was on the right side (examples of this task for both Croatian and Swedish versions can be seen in Supplementary material). A pilot study of the first version of the CDI-III-HR showed a ceiling effect on a large number of words and relatively simple syntactic structures in the sentence complexity subsection.

In November 2020, we began developing a new version using the feedback from parents and data from the pilot study. Fifty additional words were selected on the basis of the Frequency Dictionary of Croatian Children’s Language (Kuvač Kraljević et al., 2022) and added to the vocabulary subsection (such as *rugate se (mock), prosuti (spill), zijevati (yawn)*). The complexity of grammatical items was increased. For this reason, the standardised version contained 150 words in the vocabulary section, 16 items in the grammar-morphology section, and 14 sentence pairs in the syntax complexity section. In order to standardise the inventory, assessment reports were collected from parents of 620 children (311 girls and 309 boys) with typical language and cognitive development from all parts of Croatia in 2022, taking into account all dialectal language and regional cultural differences. Parents were encouraged to check off words from the list, even if the words did not match the child’s dialect. For example, if a parent of a child from the southern part of Croatia indicated the word *frigati (fry)* for the word *pržiti* (fry), the word was accepted because both words have the same meaning. The norms were developed for the two-month age range to better capture changes in lexical and grammatical development.

In the end, the CDI-III-HR was developed based on the Swedish inventory, both in terms of number of items in the vocabulary and grammatical parts, as well as the distribution of word class – nouns, verbs, and adjectives. In addition to their clinical value, these comparable formats of CDI-III offered the opportunity to conduct cross-linguistic studies covering a wide range of issues analysed from the linguistic and cultural perspectives of different languages (see for example Eriksson et al., 2012; Kuvač Kraljević et al., 2021).

The final CDI-III-HR consists of the following subsections:

- Level of communication (6 items).
- Vocabulary with a total of 100 words divided into four semantic categories: food words (16 items), body words (26 items), mental words (30 items) and emotion words (28 items).
- Grammar consists of two subsections: grammar-morphology (8 items) and syntax complexity with 10 pairs of sentences.
- Metalinguistic awareness consists of 9 items related to phonological (3 items) and orthographic (6 items) awareness.
- Pronunciation consists of 6 items; one general question and five related to the child’s ability to pronounce some sounds.

The Level of Communication subsection is not scored but has an exclusion criterion. Namely, if parents check one of the first two options (*He/she still does not speak.; He/she speaks, but his/her speech is unintelligible.*), they do not continue to fill out the inventory, as these choices indicate that the child is not yet using language to communicate. For each ticked item in the vocabulary and metalinguistic awareness subsection, the child receives 1 point. For the items in the pronunciation and grammar-morphology subsections, parents can choose between three options – *never*, *sometimes*, and *always* – and the point scale ranges from 0 to 2. In the sentence complexity subsection, the second sentence in each pair is more complex and receives 1 point.

Cronbach’s *α* for the four vocabulary categories was: food words 0.74, body words 0.88, mental words 0.93, emotion words 0.90, and for the whole vocabulary subsection 0.97. Cronbach’s *α* was 0.77 for the grammar-morphology, and 0.84 for sentence complexity. Due to the small number of items, Cronbach’s *α* was the smallest for phonological awareness (*α* = 0.39) and orthographic awareness (*α* = 0.59) as two parts of the metalinguistic awareness subsection (Kuvač Kraljević et al., in press).

### New Reynell Developmental Language Scales (NRDLS-HR)

The SLPs who participated in this study assessed the children’s language comprehension and production abilities using the Croatian version of the New Reynell Developmental Language Scales (NRDLS-HR; Edwards et al., 2019). This well-known test assesses comprehension and production of single words (nouns and verbs), morphology, and simple and complex sentences. The test has been adapted in Croatian and follows the structure of the original English version, but integrates all the peculiarities of the Croatian language, especially in the grammatical part of the test. The Comprehension Scale and the Production Scale consist of 72 items each. The test is valid for children between the ages of 2 to 7.6 years old and specific norms are available for all age groups. The norms were developed based on data collected from 791 typically developing children from different parts of Croatia and includes all dialects variations. There is a strong correlation between the Comprehension Scale and the Production Scale (Pearson correlation coefficient *r* = 0.91). The values of the reliability coefficients obtained by the split-half method (method of internal consistency) for the entire sample were 0.95 for the Comprehension Scale and 0.97 for the Production Scale. The correlation values between the two NRDLS-HR scales and the two language tests (Test for Reception of Grammar, TROG-2:HR and Peabody Picture Vocabulary Test, PPVT-III-HR) were also high, ranging from 0.74 to 0.84. The discriminant validity of the scales was verified by comparing them to a clinical sample of children with development language disorder who achieved significantly lower scores compared to children with typical language development. Measures of sensitivity and specificity were calculated. The Comprehension Scale was able to accurately identify 75% of children with a language disorder (sensitivity) and 91% of children with typical language development (specificity). The Production Scale was able to accurately identify 82% of children with a language disorder (sensitivity) and 90% of children with typical language development.

### Data analysis

A child’s ability to produce a word or combine words in syntactic structures was scored with 1 point. Although the grammar-morphology subsection offers the possibility of marking the intensity of a child’s use of some morphological forms on the scale – *never*, *sometimes*, *always* – here the categories *sometimes* and *always* are treated as one, which means that the entire subsection is scored with two values – 0 and 1. Standardised values, i.e., standardised scores and percentiles, are always used when analysing data from the NRDLS-HR because they ensure a clear classification of the individual’s performance in relation to his or her peers.

An assessment of the distribution of all three subsections – vocabulary, grammar, and metalinguistic awareness – and their eight subsections and categories – food words, body words, mental words, emotional words, grammar-morphology, syntactic complexity, phonological and orthographic awareness – showed that most of the distributions were platykurtic (i.e.) violated one of the assumptions of normality. Only three variables – vocabulary subsection and two categories: mental words, and emotional words – met the normality assumptions in all three age groups and showed symmetric distributions (*p* > 0.05). Therefore, non-parametric analyses were performed.

First, descriptive data were calculated for all subsection of the CDI-III-HR for all three age groups individually (youngest, middle and oldest). Analysis of variance (ANOVA) was conducted to examine the effects of age on a child’s language development and Spearman correlation analyses were performed to examine the associations between the different variables of the two assessment methods – parental report and formal assessment. Linear regression was used to examine whether the parental assessment of the child’s lexical, grammatical, and metalinguistic awareness knowledge predicted the child’s performance on formal language assessment. The predictor variables were tested *a priori* to check for the validity of the proportionality assumption and the absence of multicollinearity.

It is important to note that one participant in the youngest age group and two participants in the oldest age group had missing data for two variables – syntactic complexity and metalinguistic awareness. In addition, two participants from the middle age group and one participant from the oldest age group had missing data for pronunciation.

## Results

### Influence of age on language development

For all parts of the CDI-III-HR, we calculated the average performance of the children based on data collected from the parental reports.

#### Level of communication

Of the total of 51 children in the youngest age group, there were 5 children whose parents reported that they spoke two or three words, 15 children who formed complete sentences, and 31 children who produced complex sentences. In the middle age group, the parents of 11 children indicated that they spoke in complete sentences, while 31 children were able to use complex sentences. In the oldest age group, parents reported that 5 children were able to speak in complete sentences and 53 used complex sentences.

#### Pronunciation

When we considered the children in the youngest age group, four parents indicated that their children’s pronunciation sounded like that of even younger children, 26 indicated that their children sounded like their peers, and 21 indicated that they sounded somewhat more advanced than their peers. Of the total of 42 children in the middle age group, 30 parents indicated that their children sounded like most of their peers and 10 indicated that their children sound somewhat more advanced than their peers. For two children, parents did not provide any information about their pronunciation. In the oldest age group, two parents indicated that their children sounded like a younger child, 36 parents indicated that their children sounded like their peers and 19 parents indicated that their children sound somewhat more advanced than their peers. For one child, parents did not provide information about his pronunciation level.

#### Language subsection

Table 3 lists the average values for all subsections and categories of the three language variables corresponding to each age group. The mean values for all three variables increase with age, and this increase is most pronounced in relation to vocabulary and grammar. It is also evident that all three age groups show the same performance pattern – vocabulary and grammar showed better performance than metalinguistic awareness.

**Table 3:** Descriptive statistics for language subsection of the CDI-III-HR.

Subsection	Category	Age group					
		youngest (30–35 months; *n* = 51)		middle (36–41 months; *n* = 42)		oldest (42–48 months: *n* = 58)	
		Min-Max	*M* (SD)	Min-Max	*M* (SD)	Min-Max	*M* (SD)
Vocabulary	Food words (*n* = 16)	5–16	12.20 (2.40)	6–16	12.57 (2.64)	7–16	13.55 (1.88)
	Body words (*n* = 26)	8–26	17.63 (4.53)	5–26	19.21 (4.68)	14–26	21.60 (3.18)
	Mental words (*n* = 30)	2–29	15.12 (7.14)	4–30	18.60 (7.10)	10–30	22.00 (5.69)
	Emotion words (*n* = 28)	4–27	15.78 (5.17)	7–28	18.81 (5.13)	11–28	21.33 (4.66)
	Total (*n* = 100)	19–95	60.73 (17.30)	31–97	69.19 (18.11)	47–100	78.48 (13.86)
Grammar	Morphology (*n* = 8)	2–8	6.25 (1.60)	5–8	7.36 (0.91)	4–8	7.55 (0.80)
	Syntactic complexity (*n* = 10)	0–10	5.02 (3.11)	1–10	6.07 (2.85)	0–10	6.62 (2.80)
	Total (*n* = 18)	0–18	11.16 (4.51)	7–18	13.43 (3.31)	0–18	14.03 (3.56)
Metalinguistic awareness	Phonological awareness (*n* = 3)	0–3	1.51 (0.99)	0–3	1.83 (1.03)	0–3	2.02 (1.00)
	Orthographic awareness (*n* = 6)	0–5	2.55 (1.26)	1–5	2.76 (1.21)	0–6	3.60 (1.55)
	Total (*n* = 9)	0–7	4.11 (1.89)	1–8	4.60 (1.81)	0–9	5.62 (2.17)

M, Mean; SD, Standard deviation.

To investigate the effects of age on the language abilities of children as assessed by parents, a two-way ANOVA 3 × 3 was performed to understand the effect of age (youngest, middle, and oldest age groups) on lexical, grammatical, and metalinguistic abilities in children. The results show that age was statistically significant for all three variables. On lexical ability [*F* (2, 148) = 16.143, *p* < 0.000], differences were observed between all three age groups – the youngest and middle age groups (*p* = 0.048), the youngest and oldest age groups (*p* < 0.001), as well as the middle and oldest age groups (*p* = 0.021). On grammatical abilities [*F* (2, 148) = 8.159, *p* < 0.000] differences were observed between the youngest and middle age groups (*p* = 0.020), as well as the youngest and oldest age groups (*p* < 0.001) but there was no difference between middle and oldest groups (*p* = 0.740). On metalinguistic awareness [*F* (2, 148) = 8.713, *p* < 0.000] differences were observed between the youngest and oldest age groups (*p* < 0.001), as well as the middle and oldest age groups (*p* = 0.042) but not between the youngest and middle groups (*p* = 0.434).

### Interrelationships in language variables of the CDI-III-HR

Table 4 lists the correlations among the four categories of the vocabulary subsection – food word, body words, mental words and emotional words. Medium-to-large significant correlations between all categories were obtained.

**Table 4:** Correlations between categories of the vocabulary subsection.

Age group	Vocabulary category	Food words	Body words	Mental words	Emotions words
youngest (30–35 months; n = 51)	Food words	1			
	Body words	0.599^**^	1		
	Mental words	0.676^**^	0.789^**^	1	
	Emotions words	0.432^**^	0.758^**^	0.719^**^	1
middle (36–41 months; n = 42)	Food words	1			
	Body words	0.701^**^	1		
	Mental words	0.763^**^	0.749^**^	1	
	Emotions words	0.762^**^	0.767^**^	0.862^**^	1
oldest (42-48 months: n = 58)	Food words	1			
	Body words	0.658^**^	1		
	Mental words	0.666^**^	0.760^**^	1	
	Emotions words	0.654^**^	0.684^**^	0.845^**^	1

^**^Indicates a significant correlation at the 0.01 level (two-tailed).

Table 5 shows the correlation between the three language subsections of the CDI-III-HR – vocabulary, grammar and metalinguistic awareness. In all three age groups, all three variables are significantly correlated, with the highest correlation coefficients observed between vocabulary and grammar.

**Table 5:** Correlation between subsections of CDI-III-HR.

Age group	Subsection	Vocabulary	Grammar	Metalinguistic awareness
youngest (30–35 months; *n* = 51)	Vocabulary	1		
	Grammar	0.554^**^	1	
	Metalinguistic awareness	0.310^*^	0.417^**^	1
middle (36-41 months; *n* = 42)	Vocabulary	1		
	Grammar	0.694^**^	1	
	Metalinguistic awareness	0.457^**^	0.472^**^	1
oldest (42-48 months: *n* = 58)	Vocabulary	1		
	Grammar	0.547^**^	1	
	Metalinguistic awareness	0.482^**^	0.442^**^	1

^**^Indicates a significant correlation at the 0.01 level (two-tailed).

### Individual contribution of language variables derived from parental reports to the prediction of the child’s general language abilities

Before conducting the linear regression analysis, we examined the correlation between the three language variables from the CDI-III-HR – vocabulary, grammar and metalinguistic awareness – and the standardised scores on both scales of the NRDLS-HR, which were considered as a measure of general language ability.

Table 6 shows that vocabulary, grammar, and metalinguistic awareness based on the CDI-III-HR were related to language production measured using the Production Scale of NRDLS-HR, with the exception of vocabulary in the middle age group. The number of variables from CDI-III-HR, which correlated with the language comprehension measure used in the NRDLS-HR Comprehension Scale, decreased significantly with age. Comprehension was associated with vocabulary and grammar in the youngest age group, and only grammar in the middle age group. There was no association between comprehension and the three language variables in the oldest age group. Metalinguistic awareness was not related to language comprehension performance in any age group.

**Table 6:** Correlation between both scales of NRDLS-HR and all three subsections of the CDI-III-HR.

Age group	CDI-III-HR NRDLS-HR	Vocabulary	Grammar	Metalinguistic awareness
Youngest (30–35 months; *n* = 51)	Comprehension scale	0.378^**^	0.463^**^	0.273
	Production scale	0.472^**^	0.554^**^	0.435^**^
Middle (36–41 months; *n* = 42)	Comprehension scale	0.252	0.390^**^	0.085
	Production scale	0.276	0.532^**^	0.356^*^
Oldest (42–48 months: *n* = 58)	Comprehension scale	0.184	0.231	0.211
	Production scale	0.278^*^	0.400^**^	0.382^**^

^**^Indicates significant correlations at the 0.01 level; ^*^Indicates significant correlations at the 0.05 level.

Linear regression analysis was performed to test whether the children’s language performance assessed through parental reports significantly predicted their performance in formal assessment. Moreover, we wanted to examine the individual contribution of each variable of the CDI-III – lexicon, grammar, and metalinguistic awareness – to the prediction of the children’s performance on formally assessed language comprehension and production.

The results of the linear regression analysis showed that vocabulary and grammar were statistically significant in the youngest age group (30–35 months) and only grammar was significant in the middle age group (36–41 months) (Table 7). This implies that parental reports of the child’s vocabulary only up to 35 months of age and of grammar only up to 41 months of age significantly predict the child’s comprehension abilities as determined by a formal assessment, i.e., using the NRDLS Comprehension Scale.

**Table 7:** Linear regression analysis to identify the factors influencing language comprehension.

Age group	Predictors	*β*	*p*	*F*	*p*	*R*	*R^2^*	*∆R*
Youngest (30–35 months; *n* = 51)	Vocabulary	0.379	0.006	12.822	0.006	0.455	0.144	0.126
	Grammar	0.455	<0.001	8.227	<0.001	0.455	0.207	0.191
	Metalinguistic awareness	0.262	0.063	3.619	0.063	0.262	0.069	0.050
Middle (36–41 months; *n* = 42)	Vocabulary	0.265	0.090	3.022	0.090	0.265	0.070	0.047
	Grammar	0.381	0.013	6.802	0.013	0.381	0.145	0.124
	Metalinguistic awareness	0.070	0.659	0.197	0.659	0.070	0.005	−0.020
Oldest (42–48 months: *n* = 58)	Vocabulary	0.202	0.128	2.393	0.127	0.202	0.041	0.024
	Grammar	0.229	0.084	3.098	0.084	0.229	0.052	0.035
	Metalinguistic awareness	0.239	0.070	3.403	0.070	0.239	0.057	0.040

Although the contribution of the predictor variables to language production was very small, the regression analysis showed that all three predictors were statistically significant in the youngest age group, while grammar and metalinguistic awareness were statistically significant in the middle and oldest age groups (Table 8). This means that parental reports of the child’s language, especially for grammar and metalinguistic awareness, throughout the period from 30 to 48 months, significantly predict the child’s production performance as determined by formal language assessment.

**Table 8:** Linear regression analysis to identify the factors influencing language production.

Age group	Predictors	*β*	*p*	*F*	*p*	*R*	*R^2^*	*∆R*
Youngest (30–35 months; *n* = 51)	Vocabulary	0.477	<0.001	14.402	<0.001	0.477	0.227	0.211
	Grammar	0.548	<0.001	21.073	<0.001	0.548	0.301	0.286
	Metalinguistic awareness	0.390	0.005	8.786	0.005	0.390	0.152	0.135
Middle (36–41 months; *n* = 42)	Vocabulary	0.272	0.082	3.192	0.082	0.272	0.074	0.051
	Grammar	0.531	<0.001	15.702	0.001	0.531	0.282	0.264
	Metalinguistic awareness	0.389	0.011	7.135	0.001	0.389	0.151	0.130
Oldest (42–48 months: *n* = 58)	Vocabulary	0.251	0.057	3.769	0.057	0.251	0.063	0.046
	Grammar	0.315	0.016	6.184	0.016	0.315	0.099	0.083
	Metalinguistic awareness	0.356	0.006	8.123	0.006	0.356	0.127	0.111

### Agreement between language performances for children with scores in the lower range of average performance

For the final analysis, we selected only those children whose performance on the Comprehension Scale or Production Scale of NRDLS-HR was in the lowest 10% of the standard scores of the typical population of the range of the typical population (from 81 to 90 standard score). As presented in Table 2, we selected a total of 22 children (five children in the youngest age group, five in the middle age group, and 12 in the oldest age group). Since language development is still variable at the age of 3 and 4 years, the language performance of children whose achievement is near the 10th percentile (i.e., 80 standard score) is very sensitive and should be monitored. Therefore, we wanted to investigate how parents viewed the language development of these children.

**Figure 1:**
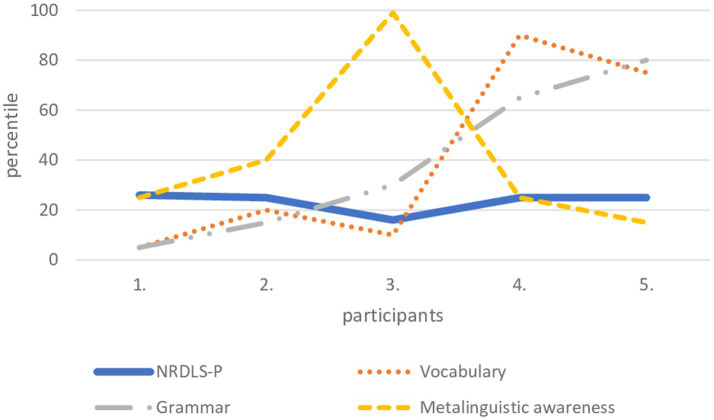
Language abilities of five children from the youngest age group obtained on the CDI-III-HR (vocabulary, grammar and metalinguistic awareness) and compared with the data obtained on the NRDLS-HR– Production scale.

Two parents of the children aged 30–35 months (fourth and fifth child depicted in Figure 1) overestimated their child’s performance in lexical knowledge and grammar. Two parents (child no. 2 and 3) rated the child’s lexical and grammatical knowledge similarly to the scores obtained on NRDLS-HR. Two parents (of child no. 2 and 3) overestimated metalinguistic awareness, which shows that this ability is difficult to assess at this age for some parents.

**Figure 2:**
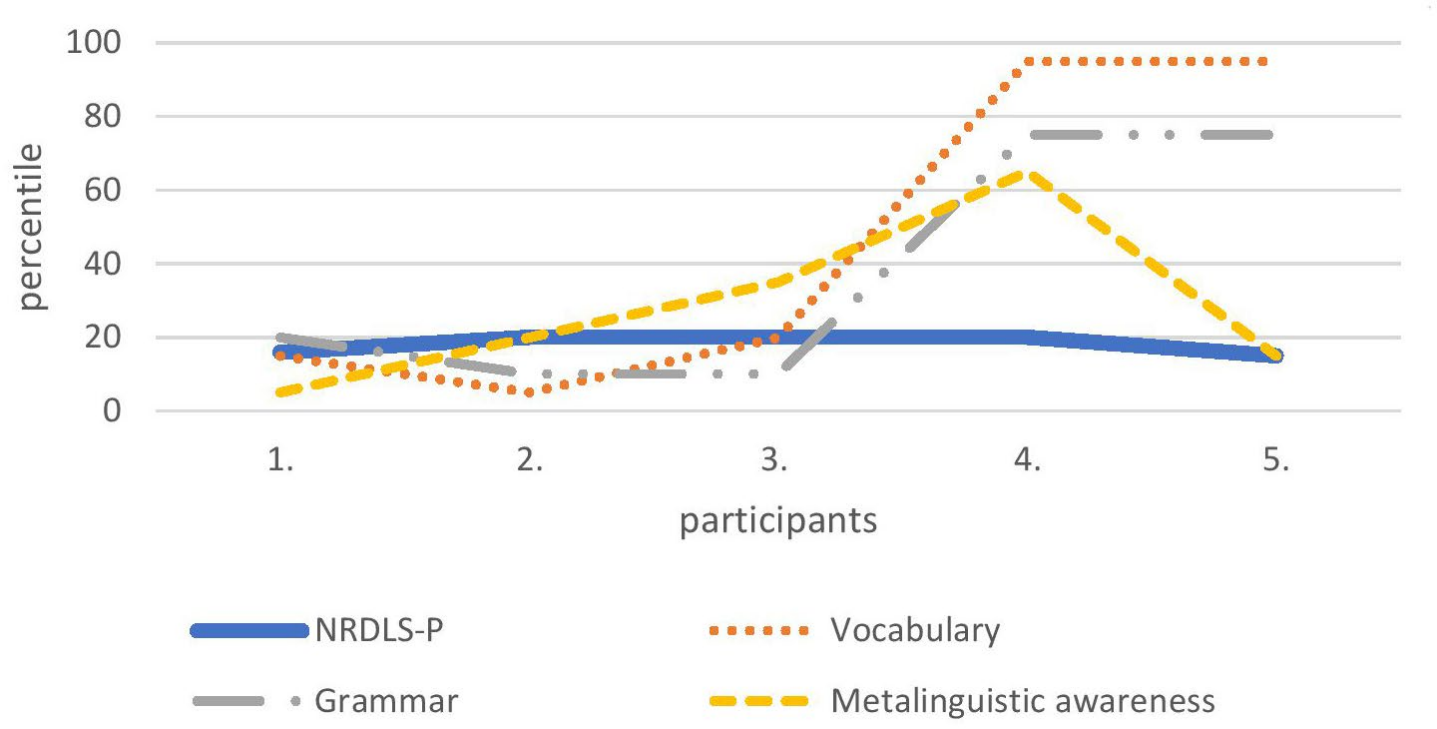
Language abilities of five children from the middle age group obtained on the CDI-III-HR (vocabulary, grammar and metalinguistic awareness) and compared with the data obtained on the NRDLS-HR– Production scale.

In the middle age group (from 36 to 41 months), two parents overestimated their child’s performance (child no. 4 and 5 in Figure 2), one parent underestimated the performance (child no. 2), and the other two parents estimated their child’s language performance similarly to the performance obtained on NRDLS-HR (child no. 1 and 3). In this age group, it was much easier for parents to assess metalinguistic awareness. In other words, parents did not overestimate this ability any more or less than they did with lexicon and grammar.

**Figure 3:**
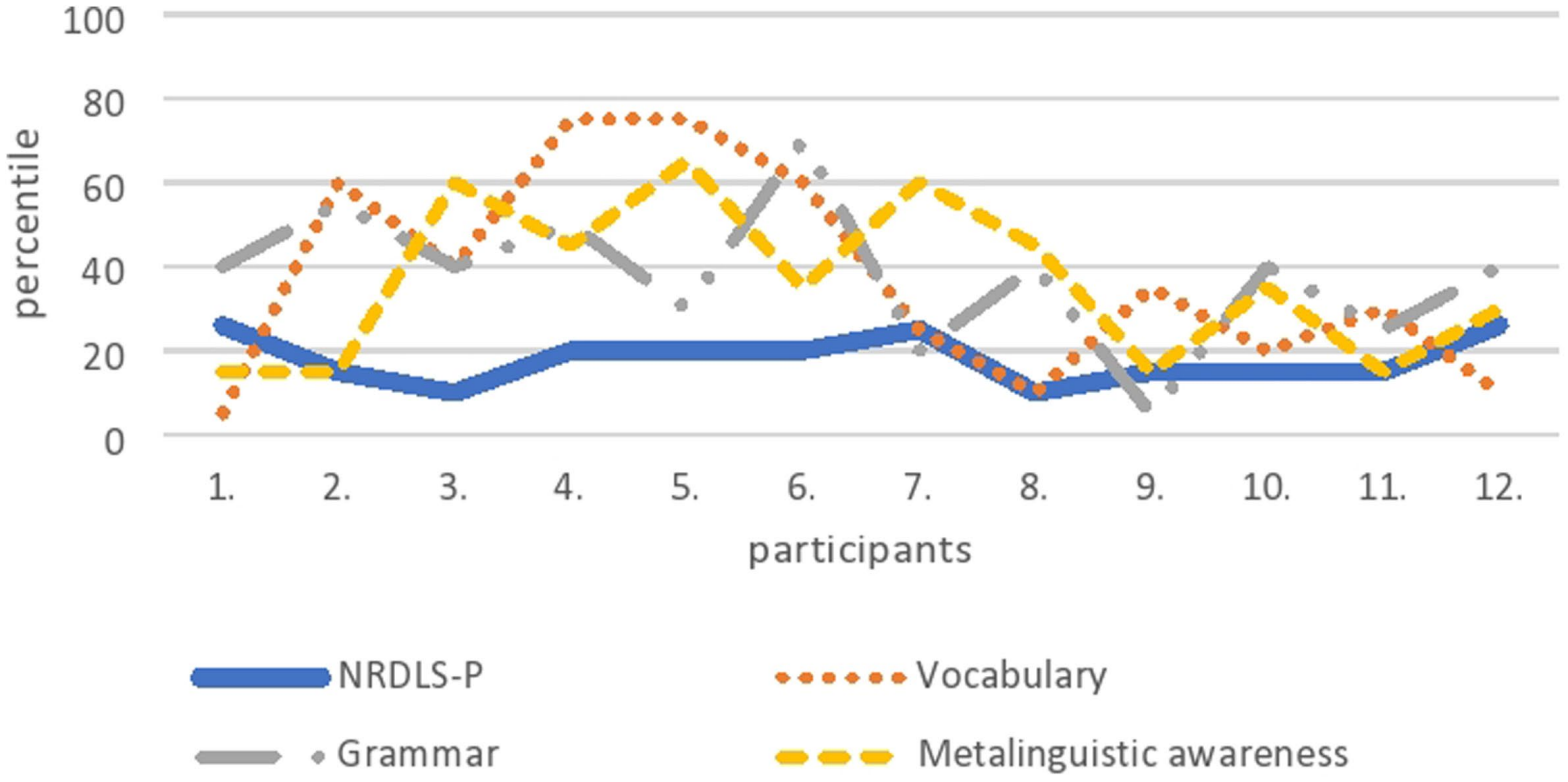
Language abilities of twelve children from the oldest age group obtained on the CDI-III-HR (vocabulary, grammar and metalinguistic awareness) and compared with the data obtained on the NRDLS-HR– Production scale.

In the oldest age group (42 to 48 months), one parent overestimated the child’s performance (child no. 1 on Figure 3) and one parent faced problems during the assessment of metalinguistic awareness (child no. 7 in Figure 3). Nearly half of the parents (*n* = 5; child no. 3 to child no. 7 in Figure 3) rated the child’s language abilities significantly higher on all three variables – lexical, grammatical, and metalinguistic awareness – such that the scores between at least one of these variables exceeded one standard deviation in comparison with the child’s performance on the NRDLS Production Scale. For the last five children (from child no. 8 to child no. 12 in Figure 3), the parents’ assessment of the children’s linguistic abilities was similar to that of the clinician.

It can be concluded that regardless of age, half of the parents were able to assess their child’s language development similarly to the scores obtained by formal language assessment. Considering the three language variables, it is challenging for parents of the youngest age group to assess metalinguistic awareness.

## Discussion

Although parental reports have proven to be an effective tool to gather information about the child’s language and communication development in the first three years of life, very little is known about the validity of parental assessments during the period when language becomes more lexically diverse and grammatically complex. Therefore, this study investigated the concurrent validity of parental reports of children between the ages 30 to 48 months by analysing and comparing the parental reports on language production abilities of typical developing children using the Croatian version of CDI-III with assessments of general language abilities.

First, the descriptive data from this study shows that, although it is a cross-sectional study, parents are able to recognise all three language abilities in a way that reflects the increase in children’s language development after 30 months. At the same time, the highest increase was observed in the lexicon and grammar, while the smallest increase was observed in metalinguistic awareness. Despite individual variations, the age factor had a significant effect on all three abilities of children’s language development, thus confirming once again that all three language abilities increase with age and that parents can perceive these developmental trajectories. In terms of the assessment method, this means that CDI-III-HR is sensitive enough to detect improvement in language development between 30 and 48 months.

When looking at lexical development based on the CDI-III-HR, it can be seen that even based on a limited and selected set of 100 words, the child’s lexical development shows linear progression across all three age groups. Although we cannot talk about the lexicon size, these results are consistent with Owens (2020), who found that a child’s vocabulary increases exponentially with age. The mean scores obtained in this study for the lexicon are similar to those obtained with the Swedish (Eriksson, 2017) and Estonian (Tulviste and Schults, 2020) versions of CDI-III. This suggests that the developed comparative formats of the CDI-III in different languages provide information about the similarity of lexical development over the period 30 to 48 months in languages that differ significantly typologically. Words are a building block for further grammatical development, so a lexicon that is not only quantitatively sufficient, but also qualitatively diverse is obligatory from the second year of childhood. Although it is slower than lexical development, the development of grammar also progresses with age, which is consistent with other studies confirming that children use more morphological rules and extend syntactic structures after the second and especially after the third year of life (Tager-Flusberg, 2001; Reed, 2017). The slowest increase was recorded in children’s metalinguistic awareness. The same is confirmed in other languages that used CDI-III such as Swedish (Eriksson, 2017) or Estonian (Tulviste and Schults, 2020). This finding is not surprising, since this ability is just beginning to develop at this age. Namely, for children at the age of three years, this is especially demanding because the implementation of metalinguistic awareness, especially phonological awareness, requires explicit linguistic knowledge about discrete language units. This type of knowledge cannot be extracted from the current communicative context (Sinclair, 1986; Whitehurst and Lonigan, 2001; Anthony and Francis, 2012).

Second, in all three age groups positive medium-to-large significant correlations were found among all four categories of the vocabulary subsection – food word, body words, mental words, and emotional words. The descriptive data show that children in all three age groups have the most words from the food and body words categories. Mental words were the least represented in the youngest age group but becoming more present after 36 months. Two explanations can be given for this: (1) the order of acquisition of certain semantic categories – it has been shown that words from the food category are acquired very early (in the second year of life) because they are an essential part of a child’s life (Eriksson, 2017). Words from the body parts category are acquired intensively between the second and third years of life as children become more familiar with their physical features. For this reason, words that describe external body parts are acquired earlier than words that describe internal parts of the body. Words from the mental words and emotions categories are acquired after the third year of life and are an extremely important part of the child’s socio-emotional development and the development of prosocial behavior (Drummond et al., 2014); (2) the concreteness of words – since the categories of food and body consist entirely of concrete words, it is reasonable to expect words of these semantic categories to be more common in the child’s early vocabulary. In the mental and emotional words categories, almost half of the words are abstract, which means they are conceptually harder for children (e.g., *believe, want, wonder*). According to Papafragou et al. (2007), a child’s vocabulary before its second year of life is defined by concrete words, while abstract words appear after the third year, which is confirmed in the present study.

By examining the relationship between the different subsections of the Croatian version of CDI-III – vocabulary, grammar and metalinguistic awareness – consistent positive moderate significant correlations were observed between the vocabulary and grammar subsections through all three age groups. The same relationship in children’s early language development up to the age of 30 months was confirmed in many other monolinguistic studies in different languages (Maital et al., 2000; Fenson et al., 2007; Stolt et al., 2009), as well as in cross-linguistic studies (Thordardottir et al., 2002; Devescovi et al., 2005; Kuvač Kraljević et al., 2021). This was also confirmed in studies where the CDI-III was employed to assess language development in childhood years after 30 months, for example, in Estonian (Tulviste and Schults, 2020) or Portuguese (Cadime et al., 2021). This suggests that the intertwining of expressive lexical skills and emerging grammar is a stable developmental pattern between the ages of three and four in children with typical language development.

Furthermore, the slowest pace of growth found in children’s language performance in metalinguistic development as reported by the parents was also perceived in low positive significant correlations between vocabulary and metalinguistic subsections in the youngest age group. This correlation becomes more and more moderate in the middle and oldest age groups, thus confirming that metalinguistic awareness is just beginning to develop at this age and will continue to increase with age. Of course, the low values of the correlation between metalinguistic and two other language measures, which are relatively constant even in the period of 30 to 48 months, indicate a different content of knowledge that lies in the background of metalinguistic knowledge related to lexicon and grammar. It is also interesting that the metalinguistic awareness subsection has consistent positive moderate significant correlations with the grammar subsection across all three age groups, unlike its correlation with vocabulary, which is the weakest in the youngest age group. Phonological awareness, as part of metalinguistic awareness, refers to the ability to detect or manipulate the phoneme in words independent of meaning (Anthony and Francis, 2012), which means that the meaning of the word is not crucial when one thinks metaphonologically, or even metaorthographically. The Croatian language is a morphologically rich language, where morphology, for example, defines the form of words or their syntactic functions. These data lead to the conclusion that the morphological form of words begins to be closely related to the explicit knowledge of the language already at an early age. This relationship between grammar and metalinguistic awareness should be investigated more comprehensively in further studies including older preschool and school age groups.

Third, the relationship between three language domains reported by parents and two formally assessed aspects of general language ability – comprehension and production – showed that language abilities assessed by parents were more closely associated with general language productive ability than comprehension. Namely, correlations between all subsections of CDI-III-HR and NRDLS-HR Comprehension Scale rapidly decreased as age increased and they completely disappeared in the oldest age group. Significant reduction in correlation strength between comprehension and production confirms the dissociation between these two aspects of language during this period of language development. A similar result was obtained by Bornstein and Hendricks (2012) at ages 36 and 47 months, in languages very similar to Croatian (such as Serbian and Bosnian). Although asymmetries in comprehension and production development are more common in early language development (Hendriks, 2014), in this study it is shown that this can be also expected in the later toddler and preschool years. These asymmetries are highly language-related and, according to Hendriks (2014), are determined primarily by the grammar. Children sometimes produce correct sentences even though they do not know their exact meaning. For example, they can produce the correct word order, and then use that sentence structure as a basis to conclude what is the object and what is the subject. This is explained as a language-as-a-signal view (Hendriks, 2014). Since languages differ from each other in their grammatical structure, not every language will have a dissociation between comprehension and production at the same stage of language development. In our study, the regression analyses further support this finding. Only vocabulary and grammar in the youngest age group and grammar in the middle age group significantly predict comprehensive language ability. Thus, this strong correlation with formally assessed comprehension abilities indicates grammatical development, which means that there is a linear progression in the child’s grammatical production observed by the parents and the child’s progression in comprehension ability. Metalinguistic awareness showed no predictive values in any age group for language comprehension, confirming once again that it corresponds to different knowledge compared to language comprehension. The conducted regression analyses support these data indicating that parental reports can predict, although at a very low variance, child lexical development in the youngest age group and for a period of 1 year with respect to grammatical development. These data contain direct clinical information, so that around the age of three, parental information about the child’s expressive grammar can be a reliable source of information for clinicians.

Significant positive low-to-moderate correlations between all three language domains of parental reports and general productive language ability were found in all three age groups, except with respect to vocabulary in the middle age group. Furthermore, there was a decrease in the correlations for vocabulary in the oldest age group and metalinguistic awareness in the middle and the oldest age group until they reached low significance. On the other hand, it is interesting to note that there is a consistent positive significant moderate correlation between the parental reports on their children’s grammatical abilities and formal language production measures across all age groups. Grammar develops more intensively between the ages of 30 and 48 months and is therefore most noticeable to parents. However, since the number of different grammatical forms in a child’s language production is not yet so great at this age, parents may notice and report all of the child’s grammatical markings. The further regression analyses confirmed inconsistent predictive role of vocabulary for general productive language abilities. While the predictor for vocabulary was no longer significant in the middle and oldest age groups, the predictor for grammar and metalinguistic awareness remained significant in all age groups. Although the contribution of all significant predictor variables was small, it can still be concluded that at this age grammar has the largest contribution. The reason for this is that this is the time when the development of grammar predominates. Indeed, up to this age, children have mainly marked one– or two–word utterances morphologically. At this age, the child begins grammatical marking at the sentence level. This also means that the child begins to apply various syntactic rules of the language. The improvement of grammatical knowledge is the reason why morphology and syntax have a greater influence on general language productive abilities after the age of three.

Finally, we wanted to see how parents rated the language performance of those children whose language performance was in the lower range of average performance measured by formal language assessment. There were several reasons for choosing this target group: first, language development at ages 3 and 4 years is still variable and it is sometimes difficult to capture all the individual characteristics of each child not just for parents, but also with standardised instruments in formal language assessment; second, the language performance of children whose performance is near the 10th percentile is very sensitive and should be monitored; and third, for these two reasons, it is obvious that it is difficult to diagnose a language development disorder at age three (Bishop et al., 2017). In our selected sample, we had five children in the youngest age group, five children in the middle age group, and 12 children in the oldest age group who scored between 81 and 90 with respect to the standard score on the NRDLS-HR. In all three age groups, the same pattern was visible: half of the parents succeeded in estimating their child’s language development similarly to the formal language assessment, 40% of them significantly overestimated their child’s language abilities, and only about 10% underestimated them. From a clinical perspective, the 10% who underestimated their child’s performance are less problematic than the 40% who overestimated their child’s performance. Namely, if clinicians rely only on parental reports in language assessment, then there would certainly be some children – among those whose language abilities were overestimated by their parents – who would enter the false negative rate, i.e., those who have language difficulties, but are recognised as children of typical language development. Thus, based on this small sample, which was used only as an example to examine the success of parental assessments of language abilities, as well as the diversity of parental assessments, it is not possible to generalise parental ability to estimate child language in any direction – even if previous studies have shown that parents can do so reliably, as claimed by Dale et al. (1989) or Guiberson et al. (2011), or that they cannot make a clear assessment at all (Law and Roy, 2008). The truth about parental ability to assess the child’s language lies somewhere in the middle – parents can be a valuable source of information about the child’s language abilities, but these reports cannot and should not be the only source of information for clinicians. Like any other assessment, assessment of child language and communication must be comprehensive and based on a variety of assessment methods (Shipley and McAfee, 2021), so parental reports can be only one of those methods.

### Limitations and further research

This study has two limitations. The first relates to the use of the NRDLS-HR as a measure for testing external validity. The test was recently standardised in Croatian and is therefore valid and reliable. However, it provides data on general language abilities, not separately on expressive lexicon, grammar, and metalinguistic awareness skills. This indicates the importance of developing separate standardised materials in Croatian to provide more reliable data on the concurrent validity of any newly developed expressive language test. The second limitation is related to sample size for the last question, which included only 22 participants. In order to make a more meaningful statement about parental ability to assess children’s language, it would be important to include more participants and expand the range of children’s performances based on formal language assessment. In other words, it would be interesting to see how parents assess children who have below-average language skills according to the formal language assessment and what patterns of parental assessment can be detected in that range of distribution. However, it would be interesting to see how parents of children with developmental language disorder perceive the language abilities of their children and how the knowledge that their child has a difficulty affects the parental image of the child’s language functioning.

Parental judgment is influenced by a number of socioeconomic factors (such as education, family income, inclusion in different social activities, and so on), as well as the personality characteristics of the parents themselves. Numerous studies have been conducted to define the role of these factors in different languages for different language measures in infant and toddler periods. Unfortunately, the results of these studies are contradictory, even when the studies were methodologically the same and conducted in the same age groups (Eriksson, 2017; Tulviste and Schults, 2020) or in younger age groups than those included in this study (Fenson et al., 1994; Berglund et al., 2005; Feldman et al., 2005; Rescorla et al., 2005; Nylund et al., 2021; Urm and Tulviste, 2021). Therefore, future research, using the CDI-III, should also consider these factors and examine their influence on parental reports in the phase of language development after the age of three.

## Conclusion

By conducting this study based on the Croatian version of the CDI-III, we aimed to contribute to the existing knowledge on the validity of parental reports of child language development after the age of 3 years. From the obtained data several important conclusions can be drawn.

First, these data contribute to the new evidence on parents’ success in assessing their child’s language in the late toddler and preschool period. In this study, parents observed the highest gains in lexicon and grammar and the lowest in metalinguistic awareness. In addition, parents observed increases in these three language skills with age, indicating that parents may perceive these developmental trajectories. Second, comparison of these data with data collected in other languages using CDI-III indicates many similarities in the timing and manner of lexical and grammatical development and development of metalinguistic awareness among languages. Third, a consistent relationship between lexical and grammatical abilities confirms that the intertwining of expressive lexical abilities and emerging grammar is a stable developmental pattern, not only in the first three years, but also between the third and fourth years of life in children with typical language development. Fourth, grammar made the largest contribution among the three predictors analysed, implying two conclusions: (a) grammar plays a prominent role in language development during this period and (b) parents may notice the child’s transition in grammar development, which can be briefly described as a transition from word grammar to sentence grammar. Fifth, the slowest rate of growth found in children’s language performance in metalinguistic development and the very low number of correlations between metalinguistic awareness and general language abilities indicate a different timing and nature of the development of this construct.

However, this study also has clinical significance. Parental reports can predict, albeit with very low variance, child lexical development up to 35 months and grammatical development up to 41 months. Therefore, parental information about the child’s language, especially expressive grammar, can be a reliable source of information for clinicians. Nevertheless, parental reports cannot be the only source of information for SLPs or other clinicians. In fact, many other formal sources of information should be considered in addition to parent reports when clinically assessing early language development.

## Data availability statement

The raw data supporting the conclusions of this article will be made available by the authors, without undue reservation.

## Ethics statement

The study and its protocol were approved by the Ethics Committee of the Faculty of Education and Rehabilitation Sciences, University of Zagreb (approval number: 251–74/22–01/2). The inclusion of SLPs in the study, the recruitment of participants at daycare centres, and the entire testing procedure were approved by the Ministry of Science and Education (MSE 533–06-21-0002). Written informed consent to participate in this study was provided by the participants’ legal guardian/next of kin.

## Author contributions

LŠB contributed substantially to the concept and design of the study, data collection, statistical data analysis, and data interpretation. JKK contributed substantially to the development of the test, the conception of the study, data interpretation and the editing of the manuscript. All authors contributed to the article and approved the submitted version.

## Funding

This study was financially supported in part by the Faculty of Education and Rehabilitation Sciences University of Zagreb and the University of Zagreb through the University Research Grant.

## Conflict of interest

The authors declare that the research was conducted in the absence of any commercial or financial relationships that could be construed as a potential conflict of interest.

## Publisher’s note

All claims expressed in this article are solely those of the authors and do not necessarily represent those of their affiliated organizations, or those of the publisher, the editors and the reviewers. Any product that may be evaluated in this article, or claim that may be made by its manufacturer, is not guaranteed or endorsed by the publisher.

## Footnotes

^1^This study is part of a larger investigation examining the reliability of parental assessment as part of the Project “Standardization of CDI-III-HR and verification of parental reliability in reporting language development of children with typical language development and children with language disorder.” Children with below-average language performance are not the subject of this paper but will be examined in a separate study.
