# Supplementary material for: Parental reports on language development in toddlers and preschoolers based on the Croatian version of Communicative Development Inventories III
Source: Front Psychol. 2023 Jul 20;14:1188550. doi: 10.3389/fpsyg.2023.1188550 (PMC10397412; doi:10.3389/fpsyg.2023.1188550)
Supplement: Supplementary file 1 [file Table_1.docx]

**Appendix: The examples of the items from grammar, sentence complexity (plus examples from the Swedish version) and metalinguistic sections of Croatian version CDI-III**

**Grammar**

|  | Nikad  [Never] | Ponekad  [Sometimes] | Uvijek  [Always] |
| --- | --- | --- | --- |
| Rabi li Vaše dijete riječi poput *više*, *još* ili *opet* u označavanju količine ili zahtijevanju? Npr. **Više** soka. Daj **još**. Hajde **opet**.  [Does your child use words like *more*, *most* or *again* to indicate quantity or demand? E.g. **More** juice. Give me **more**. Come on **again**.] |  |  |  |
| Postavlja li Vaše dijete pitanja koja počinju riječima *Tko?*, *Zašto?* ili *Kada?* Npr. **Tko** je došao? **Kada** idemo u park?  [Does your child ask questions that begin with the words *Who?,* *Why?* or *When?* E.g. **Who** came? **When** do we go to the park?] |  |  |  |

**Sentence complexity**

| Želim lutku.  [I want a doll.]  Želim lutku koja može sklopiti oči.  [I want a doll that can close her eyes.] |  |
| --- | --- |
| Imam djeda.  [I have a grandpa.]  Imam djeda, koji živi u Osijeku.  [I have a grandpa (relative) who lives in Osijek (place).] |  |

**Sentence complexity in original Swedish version**

|  | Always left | Equally often | Always right |  |
| --- | --- | --- | --- | --- |
| I want a doll. |  |  |  | I want a doll that can close her eyes. |
| I have a grandpa. |  |  |  | I have a grandpa (relative) who lives in Uppsala (place) |

**Metalinguistic awareness and early literacy**

|  | Da  [Yes] | Ne  [No] |
| --- | --- | --- |
| Vaše dijete dijeli riječi na slogove. Na primjer, kaže *ma-ma* ili *ku-ća.*  [Your child divides words into syllables. For example, he/she says *ma-ma* or *ku-ća].* |  |  |
| Vaše dijete pokazuje interes prema slovima. Na primjer, pita Vas koje je to slovo ili Vam ga pokazuje.  [Your child shows interest in letters. For example, he/she asks you which letter it is or shows it to you.] |  |  |
